# Psychological distress experienced by physicians and nurses at a tertiary care center in Lebanon during the COVID-19 outbreak

**DOI:** 10.1177/1359105321991630

**Published:** 2022-05

**Authors:** Maya Bizri, Ghida Kassir, Hani Tamim, Firas Kobeissy, Samer El Hayek

**Affiliations:** 1 American University of Beirut, Lebanon; 2 University of Florida, USA

**Keywords:** COVID-19, healthcare workers, Lebanon, mental health, psychological distress

## Abstract

The COVID-19 outbreak has had a significant mental health toll on healthcare workers in Lebanon. We examined pandemic-related psychological distress among healthcare workers in a tertiary care medical center. One hundred and fifty participants completed an online questionnaire. About half showed a high risk of acute distress (58.7%) on the GHQ-28, while most (89.3%) scored low/moderate stress on the PSS-10. The IES-R revealed concern for post-traumatic stress in one-third of participants, significantly in nurses (*p* = 0.008) and those living with vulnerable individuals (*p* = 0.030). Mental health history did not increase the risk. Our findings highlight the need for early targeted interventions during the pandemic.

## Introduction

In December 2019, the city of Wuhan in China witnessed the emergence of a novel respiratory virus, identified as the Coronavirus Disease 2019 (COVID-19). The virus rapidly spread from China to other Asian and European countries, gaining worldwide attention and causing severe public health concerns around the globe (Li et al., 2020a). In early March 2020, the World Health Organization announced the COVID-19 a global pandemic (WHO, 2020).

In addition to the stress on the healthcare system, concerns regarding the mental health impact of this disease in general and on the healthcare workforce more specifically, cannot be disregarded (Khan et al., 2020; Torales et al., 2020; Zaka et al., 2020). In China, a survey of 1257 physicians and nurses caring for patients infected with the virus showed that healthcare providers have high depressive symptoms, anxiety, insomnia, and overall distress (Lai et al., 2020). Another study on medical personnel providing direct care to patients infected with COVID-19 found alarming levels of anxiety and stress, disturbed quality of sleep, and low self-efficacy (Li et al., 2020b). One study looking at the prevalence of physical symptoms among healthcare workers in Singapore and India found that participants with reported physical symptoms were more likely to screen positive for depression, anxiety, stress, and post-traumatic stress disorder (PTSD) (Chew et al., 2020). A meta-analysis investigating the psychiatric impact of the COVID-19 pandemic on healthcare professionals showed that indirect traumatization was high among this subpopulation, exceeding psychological and emotional tolerance (da Silva and Neto, 2021).

Previous reports have examined the psychological burden experienced by healthcare workers during past outbreaks, most recently the Severe Acute Respiratory Syndrome (SARS), Middle Eastern Respiratory Syndrome (MERS), and Ebola outbreaks (Goulia et al., 2010; Ji et al., 2017; McMahon et al., 2016; Wu et al., 2009). The psychological impact included not only distress, anxiety, burnout, and somatization, but also long-term PTSD (Maunder et al., 2006). For example, a study on the impact of the 2003 SARS outbreak on medical residents in Toronto showed that worries around personal safety and risk of individual infection and the infection of loved ones conflicted with their professional duties (Rambaldini et al., 2005). Another study on the psychological impact of the MERS outbreak on healthcare workers showed that those who performed MERS-related tasks had significantly higher scores on the Impact of Events Scale-Revised (IES-R) scale, hinting toward possible PTSD (Lee et al., 2018).

Many factors play a role in the psychological distress that healthcare workers may face due to the spread of COVID-19. These include high infectivity and mortality rates, a long incubation period of the virus, concealment of information, and uncertain information about the mode of transmission and required precautions (Chen et al., 2020; O’Connor and Evans, 2020). Another factor that might burden healthcare workers is undergoing isolation and quarantine (Brooks et al., 2020), along with a growing concern about the wellbeing of family members and close contacts (Goulia et al., 2010). All of these factors add to an ongoing stressful burden of shortage in medical gears and available resources (Ranney et al., 2020). Early interventions seem to mitigate psychological distress (Maunder et al., 2008). Therefore, a call for psychological interventions to maintain the mental wellbeing of healthcare workers during this crisis has been released (De Sousa et al., 2020; Zaka et al., 2020; Zhang et al., 2020).

Lebanon is a small middle-income country located on the eastern shore of the Mediterranean Sea. The first case of COVID-19 in the country was identified on February 21, 2020, amid dire economic and financial conditions (MOPH, 2020). Previous studies only examined burnout among residents and nurses in the country, with no investigations addressing the psychological needs of healthcare workers during worldwide outbreaks (Sabbah et al., 2012; Talih et al., 2016). Therefore, we hypothesized that healthcare workers, being frontliners during the outbreak, are at a high risk of psychological distress. Understanding the psychological impact of the COVID-19 pandemic on healthcare workers in Lebanon becomes essential to implement preventive policies and effective interventions.

## Methods

### Study design and participants

This cross-sectional study was conducted between April and May 2020. A total of 800 individuals from the American University of Beirut Medical Center (AUBMC) participated. Participants were considered eligible if they were part of the medical staff at the center and belonged to one of the following categories:

- Attending medical doctors
- Clinical fellows
- Residents
- Registered nurses

Non-medical staff and research fellows were excluded from the study.

Participants were invited to fill an online self-administered questionnaire via AUB LimeSurvey, an online survey application. They were informed about the study, its purpose, and its inclusion criteria via email, followed by a link to the online survey. Individuals who were willing to participate in the survey were required to read and accept an online consent form before enrollment. A reminder of the invitation was sent three times at a four-day interval.

### Sample size calculation

As reported in a study by Wang et al. following the H1N1 pandemic, 25% of participants had a high psychological distress level on the General Health Questionnaire-28 (GHQ-28) following the outbreak (Wang et al., 2011). Assuming a type I error of 5%, a significance level at *p* < 0.05, and an absolute error or precision of 5%, the minimum sample size required to replicate this analysis in our population of interest was 147 participants. With a 20% response rate for internal surveys, a final sample size of 800 participants was obtained. Accordingly, 800 medical staff at AUBMC received the electronic questionnaire via their e-mails. A total of 174 individuals participated in the study; 150 provided complete responses that were included in the data analysis, whereas 24 participants’ results were removed due to incomplete data.

### Ethical approval

Ethical approval was obtained from the Institutional Review Board of AUBMC before the initiation of this study (SBS-2020-0152). Since the study had no foreseeable risks, consent was obtained in an electronic format. Since two questions in the survey assessed for the presence of passive death wishes and suicidal ideations, participants were provided with available resources and services to reach out for help. Individual participants also had the right to accept or refuse participation in the study, with no financial compensation provided in exchange for participation. For privacy and confidentiality, the researchers were blinded to the list of emails of participants and all data were completely de-identified.

### Questionnaire

The questionnaire included 82 questions and was administered in English since AUBMC is an English-speaking institution. It required approximately 20 minutes to be completed and was divided into six parts: demographics and personal data, data about COVID-19 exposure, psychological distress as captured by the GHQ-28, level of perceived stress as captured by the Cohen Perceived Stress Scale-10 (PSS-10), type of precautionary behaviors, and subjective distress by the traumatic event—the COVID-19 outbreak—as captured by the IES-R.

1. **Demographics and personal data:** This section included 12 questions and was used to obtain demographic information, including age, gender, marital status, living arrangement (alone, with family, with no elderly or children or people with chronic medical illness, with elderly>65, with children, or with people with chronic medical disease), and recent travel history. Participants were also asked to indicate their current status at AUBMC (profession, years since graduation, and specialty). Besides, they were asked to indicate “yes” or “no” to prompts about their mental health status: “Do you have a personal history of mental health problems,” “are you currently seeking a professional mental health provider,” and “are you currently taking any psychiatric medication?” Lastly, they were asked about their history of chronic medical illnesses.
2. **Data about COVID-19 exposure:** This section included three self-report questions of the exposure type: exposed, cared for, or quarantined (Chong et al., 2004).
3. **Psychological distress as captured by the GHQ-28:** The GHQ-28 is a self-administered questionnaire composed of 28 questions. Each question is accompanied by four possible responses, typically “not at all,” “no more than usual,” rather more than usual,” and “much more than usual.” The items provide four scores that fall into the following categories: somatic symptoms, anxiety and insomnia, social dysfunction, and severe depression (Goldberg and Hillier, 1979). The binary scoring method is used (0,0,1,1), with the two least symptomatic responses scoring “0” and the most two symptomatic responses scoring “1.” A score of more or equal to 4 is considered “positive” for the category (Goldberg and Hillier, 1979). This scale has been previously used but not validated in Lebanon (Chaaya et al., 2003).
4. **Level of perceived stress as captured by PSS-10:** The PSS-10 is a self-administered questionnaire composed of 10 questions. Four possible responses accompany each item: “0-never,” “1-almost never,” “2-sometimes,” “3-fairly often,” and “4-very often.” A score of 0–13 is low, 14–26 moderate, and 27–40 high (Cohen et al., 1983).
5. **Type of precautionary behaviors:** This section includes seven self-report questions of the different types of preventive behaviors possibly used during the COVID-19 outbreak. The behaviors are frequent hand washing, ventilating room air more frequently, wearing a mask outdoors, avoiding the outdoors, avoiding meeting people who are coughing, avoiding meeting people with confirmed infection, and reducing the use of public transport (McAlonan et al., 2007).
6. **Subjective distress secondary to the COVID-19 outbreak as captured by the IES-R:** The IES-R is a self-administered questionnaire composed of 22 questions. Five possible responses accompany each item: “0-not at all,” “1-a little bit,” “2-moderately,” “3-quite a bit,” and “4-extremely.” Responses are grouped into three subscale scores for intrusion, avoidance, and hyper-arousal. A total score of 24 or more raises clinical concern for PTSD, whereas a score of 33–38 represents the best cutoff for a probable diagnosis of PTSD (Weiss and Marmar, 1997).

### Statistical analysis

Descriptive statistics were summarized by presenting the numbers and percentages for categorical variables. The associations between each of the outcomes (GHQ-28, PSS-10, and IES-R) and categorical variables were assessed by the Pearson Chi-square test or Fisher’s exact test, as appropriate. Moreover, multivariate stepwise logistic regression was carried out to identify the predictors of GHQ-28, where the *p*-value for entry was 0.3, and that for removal was 0.4. Results were presented as adjusted odds ratios (aOR) with 95% confidence intervals (CI). A *p*-value of less than 0.05 was used to indicate statistical significance. We used IBM SPSS statistical software for Windows version 22 (SPSS for Windows, version 22; SPSS, Inc., Chicago, IL, USA).

### Data sharing statement

The current article includes the complete raw dataset collected in the study including the participants’ data set, syntax file, and log files for analysis. Pending acceptance for publication, all of the data files will be automatically uploaded to the Figshare repository.

## Results

### Sample characteristics

A total of 800 electronic questionnaires were sent via e-mail: 174 individuals participated in the study and 150 completed it (18.75%). The sample consisted of 66 males (44%) and 84 females (56%). These included 54 post-graduate trainees (36%), 34 senior attending physicians (22.7%), 56 registered nurses (37.3%), and 6 clinical fellows (4%). Participants were specialized in internal medicine (21.3%), pediatrics (13.3%), surgical specialties (12.7%), emergency medicine (11.3%), family medicine (5.3%), anesthesiology (4%), obstetrics and gynecology (2%), or other specialties (27.3%). More than half of the participants had less than 5 years of experience (56.4%). The majority reported no recent travel history (85.2%), no history of mental health problems (81.3%), and no history of chronic medical problems (83.2%). Other demographic variables are presented in Table 1.

**Table 1.** Sociodemographic characteristics of the sample of participants.

Demographics		*N* (%)
		*N* = 150
Gender	Male	66 (44.0)
	Female	84 (56.0)
Age	<30	86 (57.7)
	30–35	28 (18.8)
	>35	19 (12.8)
	36–50	16 (10.7)
Marital status	Single/divorced	98 (66.2)
	Married	50 (33.8)
Profession	Post-graduate trainee/clinical fellow/senior attending physician	94 (62.7)
	Registered nurse	56 (37.3)
Years of experience	⩽5	84 (56.4)
	>5	65 (43.6)
Specialty of work	Family medicine/emergency medicine/anesthesia	31 (21.2)
	Others	115 (78.8)
Living arrangement	Living with no elderly, children, or individual with chronic medical illness	79 (54.5)
	Living with elderly, children, or individual with chronic medical illness	66 (45.5)
Recent travel history	Yes	22 (14.8)
History of mental health problems	Yes	28 (18.7)
History of chronic medical problems	Yes	25 (16.8)

### Data about COVID-19 exposure and related precautionary behaviors

Table 2 shows that 32% of the participants were exposed to COVID-19, 28% were taking care of patients with COVID-19, and 44% were quarantined during COVID-19. More than half of the participants were classified as involved in precautionary behaviors.

**Table 2.** Pattern of COVID-19 exposure and related precautionary behaviors in the sample of participants.

COVID-19 related information	*N* (%)
	*N* = 150
Exposure to COVID-19	48 (32.0)
Taking care of patients with COVID-19	42 (28.0)
Quarantined during COVID-19	66 (44.0)
Precautionary behaviors	*N* (%)
Frequently washing hand	145 (96.7)
Avoiding outdoors	117 (78.0)
Ventilating room air more frequently	107 (71.3)
Avoiding meeting with people who are coughing	143 (95.3)
Wearing masks outdoors	115 (76.7)
Reducing the use of public transportation	143 (95.3)
Avoiding meeting people with confirmed infection	143 (95.3)

### Psychological distress and psychiatric symptoms

Table 3 presents the prevalence of psychological distress and psychiatric symptoms among participants. About half showed a high risk of acute distress (58.7%) on the GHQ-28, while most participants (89.3%) scored low/moderate stress on the PSS-10. The IES-R revealed no concern for PTSD among the majority of participants (70%).

**Table 3.** Prevalence of psychological distress and psychiatric symptoms in the sample of participants as per the GHQ-28, the PSS-10, and IES-R.

Scales		*N* (%)
		*N* = 150
GHQ-28	Low risk of acute distress	62 (41.3)
	High risk of acute distress	88 (58.7)
PSS-10	Low/Moderate stress	134 (89.3)
	High stress	16 (10.7)
IES-R	No concern for PTSD	105 (70.0)
	Clinical concern/probable diagnosis/highly suggestive of PTSD	45 (30.0)

### Between-group analyses, associated factors, and subscales

Females, participants who are not married, and those who have less than 5 years of experience scored higher on the three scales, with a significant difference between males and females on the GHQ-28 (*p* = 0.025). Also, there was a significant difference among physicians and nurses at the level of subjective distress on the IES-R (*p* = 0.008), where nurses showed a higher level of clinical concern for PTSD (53.3%). Individuals living with children, the elderly, or people with chronic illness had a significantly higher level of clinical concern for PTSD (*p* = 0.030) compared to individuals who lived with non-vulnerable people. Interestingly, participants with recent travel history, mental health history, a medical illness, and exposure to COVID-19 had lower scores on psychological distress, perceived stress, and concern for PTSD as compared to their counterparts, with a significant difference only among participants with mental health history (*p* = 0.035) on the IES-R scale. Also, participants with or without mental health history had equal levels of acute distress (almost 50%) on the GHQ-28 scale (Table 4).

**Table 4.** Analysis of results on the GHQ-28, PSS-10, and IES-R in the sample of participants, stratified by the different sociodemographic variables.

Variable	Scale								
	GHQ-28			PSS-10			IES-R		
	Low risk	High risk	*p*-value	Low/Moderate	High	*p*-value	No concern	Clinical concern	*p*-value
	*N* = 62	*N* = 88		*N* = 134	*N* = 16		*N* = 105	*N* = 45	
Gender			**0.025**			0.119			0.085
Male	34 (54.8)	32 (36.4)		62 (46.3)	4 (25.0)		51 (48.6)	15 (33.3)	
Female	28 (45.2)	56 (63.6)		72 (53.7)	12 (75.0)		54 (51.4)	30 (66.7)	
Age			0.642			0.165			0.796
<30	32 (51.6)	54 (62.1)		78 (58.6)	8 (50.0)		58 (55.2)	28 (63.6)	
30–35	13 (21.0)	15 (17.2)		22 (16.5)	6 (37.5)		20 (19.0)	8 (18.2)	
>35	9 (14.5)	10 (11.5)		17 (12.8)	2 (12.5)		15 (14.3)	4 (9.1)	
36–50	8 (12.9)	8 (9.2)		16 (12.0)	0 (0.0)		12 (11.4)	4 (9.1)	
Marital status			0.623			0.267			0.276
Married	22 (36.1)	28 (32.2)		43 (32.3)	7 (46.7)		38 (36.5)	12 (27.3)	
Others	39 (63.9)	59 (67.8)		90 (67.7)	8 (53.3)		66 (63.5)	32 (72.7)	
Profession			0.155			0.786			**0.008**
Post-graduate trainee/clinical fellow/senior attending physician	43 (69.4)	51 (58.0)		83 (61.9)	11 (68.8)		73 (69.5)	21 (46.7)	
Registered nurse	19 (30.6)	37 (42.0)		51 (38.1)	5 (31.3)		32 (30.5)	24 (53.3)	
Years of experience			0.513			0.425			0.894
⩽5 years	33 (53.2)	51 (58.6)		73 (54.9)	11 (68.8)		59 (56.7)	25 (55.6)	
>5 years	29 (46.8)	36 (41.4)		60 (45.1)	5 (31.3)		45 (43.3)	20 (44.4)	
Specialty			0.984			1.000			0.067
ED/Anesthesia/FM	13 (21.3)	18 (21.2)		28 (21.4)	3 (20.0)		26 (25.2)	5 (11.6)	
Others	48 (78.7)	67 (78.8)		103 (78.6)	12 (80.0)		77 (74.8)	38 (88.4)	
Living with			0.698						**0.030**
Child/elderly/chronic illness	28 (47.5)	38 (44.2)		58 (45.0)	8 (50.0)	0.793	40 (39.6)	26 (59.1)	
None of the above	31 (52.5)	48 (55.8)		71 (55.0)	8 (50.0)		61 (60.4)	18 (40.9)	
Travel history	7 (11.3)	15 (17.2)	0.313	17 (12.8)	5 (31.3)	0.063	17 (16.2)	5 (11.4)	0.449
Mental health history	10 (16.1)	18 (50.5)	0.503	24 (17.9)	4 (25.0)	0.501	15 (14.3)	13 (28.9)	**0.035**
Medical illness	11 (17.7)	14 (16.1)	0.790	23 (17.3)	2 (12.5)	1.000	17 (16.2)	8 (18.2)	0.767
COVID-19 exposure	22 (35.5)	26 (29.5)	0.443	41 (30.6)	7 (43.8)	0.395	36 (34.3)	12 (26.7)	0.359
Care for COVID-19 patients	18 (29.0)	24 (27.3)	0.813	36 (26.9)	6 (37.5)	0.385	31 (29.5)	11 (24.4)	0.525
Home quarantine	28 (45.2)	38 (43.2)	0.810	57 (42.5)	9 (56.3)	0.425	49 (46.7)	17 (37.8)	0.315

*Note:* Bold values denote statistical significance at the *p* < 0.05 level.

In terms of subscales, there was a significant difference between physicians and nurses on the social dysfunction subscale of the GHQ-28, with the latter scoring higher (*p* = 0.020). Participants with a mental health history and those who followed up with mental health providers showed significantly different results for all the GHQ-28 subscales (*p* = 0.041 for anxiety and insomnia, *p* = 0.030 for social dysfunction, *p* = 0.058 for severe depression, and *p* = 0.004 for chronic distress). Lastly, patients with medical illness had significantly higher scores on the depression subscale compared to their healthy counterparts (*p* = 0.002). On the IES-R, females and nurses scored more significantly on intrusion and avoidance subscales (*p* = 0.053 and *p* = 0.041 for intrusion, *p* = 0.013 and *p* = 0.007 for avoidance, respectively). Also, physicians from the emergency department, anesthesia, and family medicine had a significantly lower score for the intrusion and hyperarousal subscales (*p* = 0.014 and *p* = 0.032, respectively). Finally, participants who were not living with vulnerable individuals had significantly less intrusive experiences than participants who were (*p* = 0.013). Participants with a mental health history and following up with a mental health advisor had significantly better scores for the three IES-R subscales (0.000 <*p* < 0.038).

### Multivariate logistic regression analyses

Multivariate stepwise logistic regression analyses were conducted to identify the predictors of GHQ-28. The results are presented in Table 5 and Supplementary Tables 1 and 2. Participants who had a high score on GHQ-28 were three times more likely to have higher levels of perceived stress on PSS-10 (OR = 3.21; CI [0.60–17.05]) (*p* = 0.170) and six times more likely to have concern for PTSD as captured by IES-R (OR = 6.49; CI [2.38–17.67]) (*p* < 0.0001). The model also showed that people with a recent travel history were two times more likely to have a high score on GHQ-28 (OR = 2.04 CI [0.69–6.10]) (*p* = 0.200).

**Table 5.** Stepwise multivariate logistic regression for GHQ-28, including both PSS-10 and I-ESR.

GHQ-28 (reference: low)				
Variable	aOR	95%CI		*p*-value
		Lower	Upper	
Living arrangement (reference: none)	0.61	0.28	1.34	0.210
Travel history	2.04	0.69	6.10	0.200
PSS-10 (reference: low)	3.21	0.60	17.05	0.170
IES-R (reference: no concern)	6.49	2.38	17.67	**<0.0001**

Note: Bold values denote statistical significance at the *p* < 0.05 level.

## Discussion

This study aimed to understand the psychological impact of the COVID-19 pandemic on healthcare workers in Lebanon to effectively implement policies and interventions. This is of interest as a recent systematic review and meta-analysis of 33,062 participants showed high scores for depression (22.8%) and anxiety (23.2%) among healthcare workers during the outbreak (Pappa et al., 2020). The underlying factors causing anxiety, depression, and stress were mainly increased working hours and lower psychological and logistic support (Elbay et al., 2020). Continuous psychological distress related to the pandemic is expected to have a deleterious impact, not only on mental wellbeing but also on physical health. Indeed, it was shown that workplace stressors are related to long-term effects on physical health such as cardiometabolic risk (Melamed et al., 2006). Stress also disturbs the body’s physiological stress response system causing further deterioration (Chang, 2019). In this study, we hypothesized that the Lebanese healthcare workers will have high levels of psychological distress. We expected this to be additionally fueled by the economic instability and sociopolitical turmoil that the country has been facing over the past year. Indeed, a considerable number of participants had a high risk of acute distress on GHQ-28. However, the majority of participants had low/moderate stress as measured by the PSS-10 and no concern for PTSD as captured by IES-R.

To start with, female participants showed higher acute stress and intrusion and avoidance symptoms compared to males. This comes along with the results of several recent studies describing heightened psychological distress in females during pandemics (Giordani et al., 2020; González-Sanguino et al., 2020; Zhang and Ma, 2020).

When it comes to the medical profession, nurses had higher concern for PTSD and scored worst on several subscales of psychological distress and trauma, including social dysfunction and intrusion and avoidance, respectively. Kramer and colleagues had similar results, with nurses reporting higher subjective burden and stress than doctors and other hospital staff (Kramer et al., 2020). This can be explained by several factors; nurses are expected to be in closer and more frequent contact with COVID-19 positive patients, which might contribute to an added burden. In addition, nurses typically have a higher level of work-related burnout compared to other medical professionals (Chou et al., 2014). In Lebanon, particularly, a recent study conducted at AUBMC showed very high rates of burnout and depression in nurses, reaching 52.7% and 36.2% respectively (Talih et al., 2018). Alternatively, when looking into specialties, physicians from the emergency department, family medicine, and anesthesia showed less distress and no concern for PTSD as compared to other subgroups. These specialties have been shown to endure high psychological distress (Ali et al., 2020; Rodriguez et al., 2020) due to several factors, including the frequent exposure to patients with COVID-19 infection and having subsequently fear of acquiring the disease and transmitting it to their loved ones. Our unexpected results of low distress in these critical specialties might be explained by the low sample size in each category and not including otolaryngology, another highly critical specialty, as a separate group but rather putting it within the surgical specialty category. Otherwise, the results can be explained by the extensive training done for these high-risk groups at AUBMC and the continuous support provided for them during the pandemic (AUBMC, 2020).

When looking into the living situation of participants, those residing with vulnerable populations (children, elderly, or people with chronic illness) showed higher levels of clinical concern for PTSD. Also, patients with a chronic medical illness had more elevated scores on the depression subscale compared to their healthy counterparts. Indeed, vulnerable populations are more likely to experience a debilitating COVID-19 illness course (Kadambari et al., 2020; Sanyaolu et al., 2020; Sciacqua et al., 2020) and subsequently worst mental health outcomes (Flint et al., 2020; Kang et al., 2020; Xiong et al., 2020; Webb, 2020). Individuals catering to their needs, particularly healthcare workers, would also be expected to experience high levels of psychological distress due to fear of transmitting the disease to them. Social support is, therefore, needed in both groups to provide emotional and psychological comfort during the pandemic (Zysberg and Zisberg, 2020). This is of relevance to Lebanon, where the accompanying economic deterioration, dire resources, and lack of access to medical services further adds to the burden (Arafat et al., 2020; Khoury et al., 2020).

When looking at previous mental health history, most participants showed no psychological distress or clinical concern for PTSD. One would expect that those with a mental health problem would be more vulnerable to stress. In fact, recent studies have shown that the current pandemic affected individuals with psychiatric disorders negatively, with more endured challenges and higher levels of psychological distress as compared to the general population (Druss, 2020; Iasevoli et al., 2020; Shinn and Viron, 2020). However, one can argue that those with pre-existing psychiatric disorders are more prepared to deal, accept, and adapt to stressful situations. Underreporting of symptoms can provide another explanation for the observed results, particularly in a country with a high level of stigma toward mental health in the general population (Abi Doumit et al., 2019). Moreover, healthcare workers tend to have lower rates of seeking help and receiving treatment due to embarrassment or confidentiality concerns (Cai et al., 2020), potentially further contributing to the underreporting of symptoms. Besides, a recent study examined how individuals with different classes of pre-existing mental health problems (anxiety-related vs mood-related) react to and cope with COVID-19 (Asmundson et al., 2020). Results showed a difference in stress symptoms depending on the pre-existing mental illness (Asmundson et al., 2020). As such, further exploration of the association between specific psychiatric diagnoses and mental health outcomes during the COVID-19 pandemic might be warranted.

Lastly, higher scores of acute stress were found to be associated with heightened clinical concern for perceived distress and PTSD. This suggests the importance of early detection of symptoms and the implementation of effective treatment strategies to avoid the development of more severe psychological sequelae (Arora and Grey, 2020; Matias et al., 2020; Pappa et al., 2020), particularly in low and middle-income countries (De Sousa et al., 2020). A phased model of mental health burden and responses targeted toward mental health professionals has been proposed (Tomlin et al., 2020). In France, for instance, a regional group of 39 hospitals offered a mental health hotline accessible to all hospital workers (Geoffroy et al., 2020). Such work is yet to be done in Lebanon. This is especially true in the setting of the Beirut Blast that has compounded the effect of COVID-19 related stressors on healthcare workers and only worsened the paucity of mental health resources and manpower (El Hayek and Bizri, 2020).

This study has several limitations. First, its cross-sectional design and small sample size limited the power of the statistical analysis. In addition, participants belonged to one institution limiting the generalization of results to healthcare workers in other medical institutions in the country. At AUBMC, the interaction with patients with COVID-19 was initially limited to the flu clinic, the emergency department, and an inpatient unit. The latter had a limited number of patients at the time of the conduction of the study, possibly undermining our results. Future studies would benefit from including a larger sample of healthcare workers belonging to different hospital settings. One additional disadvantage is the presence of a current political turmoil and economic crash in the country, which started before the pandemic and worsened after it. This might have acted as a confounder and accounted for a partial deterioration in the mental health status of the population in general, and our participants in particular. Finally, the scales used in this study have not been previously validated in Lebanon. The authors addressed this limitation by providing a variety of scales to account for the different aspects of mental illness.

In conclusion, this study is the first to assess the impact of COVID-19 on mental health among healthcare workers in Lebanon. Most results parallel those of other global studies that highlight the need for early targeted interventions toward vulnerable populations, including medical professionals. These are not frontliners specific, but also include less COVID-19 exposed specialties, nurses, those living with at-risk populations, and those suffering from chronic medical illnesses. This is of utmost urgency in Lebanon in light of the unstable economic status and the recent Beirut blast. Further studies are needed to examine the type of intervention needed taking into account the cultural and national implications at play.

## Supplemental Material

sj-pdf-1-hpq-10.1177_1359105321991630 – Supplemental material for Psychological distress experienced by physicians and nurses at a tertiary care center in Lebanon during the COVID-19 outbreak Click here for additional data file. Supplemental material, sj-pdf-1-hpq-10.1177_1359105321991630 for Psychological distress experienced by physicians and nurses at a tertiary care center in Lebanon during the COVID-19 outbreak by Maya Bizri, Ghida Kassir, Hani Tamim, Firas Kobeissy and Samer El Hayek in Journal of Health Psychology
