# Supplementary material for: Psychological distress experienced by physicians and nurses at a tertiary care center in Lebanon during the COVID-19 outbreak
Source: J Health Psychol. 2021 Feb 10;27(6):1288–300. doi: 10.1177/1359105321991630 (PMC7879044; doi:10.1177/1359105321991630)
Supplement: sj-pdf-1-hpq-10.1177_1359105321991630 – Supplemental material for Psychological distress experienced by physicians and nurses at a tertiary care center in Lebanon during the COVID-19 outbreak [file sj-pdf-1-hpq-10.1177_1359105321991630.pdf]

Supplementary Tables

Supplementary table 1.

a) Stepwise multivariate logistic regression for GHQ-28 without including PSS-10 and IESR.

GHQ-28 (reference: low)				
	**OR**	**95% C.I.**		**P-value**
		**Lower**	**Upper**	
Gender (reference: male)	1.49	0.74	3.00	0.27
Profession (reference: Post graduate trainee/ Clinical fellow/ Senior attending physician)	1.51	0.71	3.21	0.29
Travel history	2.15	0.76	6.09	0.15

b) Stepwise multivariate logistic regression for GHQ-28 including PSS-10 only.

GHQ-28 (reference: low)				
	**OR**	**95% C.I.**		**P-value**
		**Lower**	**Upper**	
Profession (reference: Post graduate trainee/ Clinical fellow/ Senior attending physician)	1.78	0.83	3.80	0.14
Travel history	1.97	0.68	5.75	0.21
COVID exposure	0.64	0.30	1.38	0.25
PSS-10 (reference: low)	5.12	1.06	24.79	**0.04**

c) Stepwise multivariate logistic regression for GHQ-28 including IESR only.

GHQ-28 (reference: low)				
	**OR**	**95% C.I.**		**P-value**
		**Lower**	**Upper**	
Living arrangement (reference: none)	0.64	0.30	1.37	0.25
Travel hx	2.35	0.81	6.83	0.11
IESR (reference: no concern)	7.04	2.63	18.88	**<0.0001**

Supplementary table 2.

a) Association between PSS-10 and IESR.

		IESR categories		
		No concern for PTSD	Clinical concern/Probable diagnosis of PTSD/Suppressed immune system	P-value
**PSS-10 categories**	Low/Moderate stress	99 (94.3)	35 (77.8)	**0.005**
	High stress	6 (5.7)	10 (22.2)	

b) Association between PSS-10 and GHQ-28.

		GHQ-28 categories		
		Low risk of acute distress	High risk of acute distress	P-value
**PSS-10 categories**	Low/Moderate stress	60 (96.8)	74 (84.1)	**0.01**
	High stress	2 (3.2)	14 (15.9)	

c) Association between IESR and GHQ-28.

		GHQ-28 categories		
		Low risk of acute distress	High risk of acute distress	P-value
**IESR categories**	No concern for PTSD	56 (90.3)	49 (55.7)	**<0.0001**
	Clinical concern/ Probable diagnosis of PTSD/Suppressed immune system	6 (9.7)	39 (44.3)	
